# Association between medicine Price declaration by pharmaceutical industries and retail prices in Malaysia’s private healthcare sector

**DOI:** 10.1186/s40545-019-0176-z

**Published:** 2019-07-03

**Authors:** Nur Sufiza Ahmad, Ernieda Hatah, Mohd Makmor-Bakry

**Affiliations:** 1 Faculty of Pharmacy, Universiti Kebangsaan Malaysia, Jalan Raja Muda Abdul Aziz, 50300 Kuala Lumpur, Malaysia; 2 Pharmaceutical Services Division, Ministry of Health Malaysia, 46350 Petaling Jaya, Selangor Malaysia

**Keywords:** Transparency, Pharmaceutical pricing, Drugs, Affordability, Price information, Policy

## Abstract

**Background:**

As part of the initiatives to increase price transparency for consumers, pharmaceutical industry in Malaysia have been encouraged to declare the wholesale and recommended retail prices (RRP) of medicines to the Pharmaceutical Service Department (PSD) yearly. However, the relationship between the voluntary price reporting practices and consumers’ retail medicine price is unknown. Therefore, this study aims to evaluate the effect of the voluntary price reporting practice of pharmaceutical industry on retail medicine prices, factors that may affect consumer medicine prices in Malaysia’s private healthcare sector, and the retail medicine pricing trend over 2011–2015.

**Methods:**

A yearly correlation test for a 5-year period was performed to investigate the association between the wholesale and RRP medicine prices declared by the pharmaceutical industry from 2011 to 2015 on the one hand and the consumer wholesale and retail medicine price database on the other hand. The median price ratio (MPR) was calculated by comparing the consumer retail medicine price to its international reference price. The Krukal Wallis test was used to analyse the pricing trend throughout the 5-year period, and factors that might elevate the MPR above 2.5 were modelled using binary logistic regression.

**Results:**

A total of 2527 medicine price data were analysed. There was a strong significant association between medicine prices declared to the PSD and the retail medicine prices in every year of the 5-year period. Moreover, there was no significant increase in retail medicine prices throughout the 5-year period. The medicine types, retail location, type of manufacturer, medicinal indications, declared wholesale and RRPs significantly influenced the consumer MPRs that where > 2.5.

**Conclusion:**

The declared medicine price was found to have a significant association with the consumer retail medicine price. Thus, it may be a useful reference for consumers purchasing medicines in private healthcare settings. However, the government of Malaysia must develop strategies to increase medicine price transparency for price-control mechanisms in the private healthcare sector.

**Electronic supplementary material:**

The online version of this article (10.1186/s40545-019-0176-z) contains supplementary material, which is available to authorized users.

## Background

The Malaysian healthcare system consists of public and private healthcare providers, who are independent of each other. The public healthcare sector, which is the main provider of healthcare services in Malaysia, is funded through the Consolidated Revenue Fund managed by the Ministry of Finance, while the private healthcare sector is funded by consumer out-of-pocket payments, private health insurance, and employer benefit schemes [1]. Government tax exemptions and incentives are only granted to hospitals that provide medical tourism [2]. The ratio of healthcare spending between the public and private sectors was reported as 52:48 [3]. In 2014, healthcare expenditure in Malaysia was RM 49.73 billion (USD 15.64 billion), with private spending accounting for RM 23.92 billion (USD 7.52 billion) [4]. In public healthcare facilities, patients are only charged a nominal fee of RM 1 (USD 0.31) to RM 5 (USD 1.57) for each outpatient visit, while the fees in private healthcare facilities are usually higher [5]. In the public healthcare sector, containment of medicine prices is achieved by bulk purchase through concession supply and national tender. Prices are usually set using internal and external reference pricing mechanisms [5]. These pricing mechanisms involve comparing the price with other medicines in the same therapeutic group, or comparing purchase prices in public healthcare institutions with the international reference prices (IRP) [5]. Conversely, in the private healthcare sector, only the doctor’s consultation fees are regulated by the government. Thus, the prices of medicines in the private healthcare sector are reported to be variable and highly augmented [6].

A previous study by Babar et al. [6] in Malaysia regarding the pricing of 48 medicines in 32 community pharmacies and 20 dispensing doctors showed that the prices of innovator brands (IB) and generic medicines were at least 15 and 6.6 times higher, respectively, than their international reference prices (IRPs). The price markup in dispensing doctors’ clinics ranged between 50 and 76% for IBs, and could be up to 316% for generics. In retail pharmacies, the markup price ranged between 25 and 38%, and 100 and 140% for IBs and generics, respectively [6]. In another study, the medicine prices in retail pharmacies in the northern region of Malaysia were found to be higher than the mean retail price in Australia [7]. The high prices of medicines may reduce accessibility and affordability, which may affect the consumer’s decision to purchase medicines, leading to poor healthcare quality and irrational use of medicines [8].

There is, therefore, a need for the Malaysian government to structure a scheme for rational pricing of medicines in the private healthcare sector. As part of the initiative to ensure adequate, continuous, and equitable access to high-quality, safe, effective, and affordable medicines, Malaysia’s Ministry of Health (MOH) has developed a National Medicine Policy (MNMP) that encourages initiatives to increase transparent medicine pricing in Malaysia, and provide consumers with relevant and readily available information [9]. Hence, in 2011, the MOH began encouraging pharmaceutical companies to voluntarily declare their wholesale and recommended retail price (RRP) to the Pharmaceutical Service Division (PSD) [9]. The declared prices were then used to develop the RRP database that was published on the PSD website, to be used as a guide during medication purchasing by consumers [9]. The declared wholesale prices were used only for monitoring purposes by the PSD and were not made available to the public. This initiative has previously been adopted by Canada and several European countries such as Austria, Bulgaria, Cyprus, Lithuania, and France, who requested their pharmaceutical industries to declare ex-factory drug prices to the government, which are then used during price negotiations [10, 11]. Price transparency was reported to provide consumers with options, thus allowing for more efficient and effective procurement [12]. In the long term, it may lead to reduce medicine prices through healthy competition, thus increasing consumer access to affordable medicines.

In Malaysia, although the voluntary reporting practice has been implemented for several years, no study has evaluated how the practice influences retail medicine prices. Thus, we investigated: 1) the association between medicine prices declared to the PSD and retail prices, 2) factors that may influence the construct of retail medicine prices in the private healthcare setting in Malaysia, and 3) the retail medicine price trend from 2011 to 2015.

## Methods

The investigations were performed using the PSD medicine price database from 2011 to 2015, which consisted of: 1) the wholesale and RRP prices of controlled and over-the-counter medicines, as declared by pharmaceutical companies, and 2) consumer wholesale and retail medicine prices (sampled by the PSD through the Medicine Price Monitoring Survey (MPMS) at institutional counters or from medicine invoices, or both) [13]. The MPMS reviewed prices of medicines commonly used for acute and chronic diseases (consisting of 25 core and 32 supplementary medicines), listed by the World Health Organization (WHO). The national MPMS is conducted yearly following the recommended methodology of the WHO for “baskets of medicines” in the public and private healthcare sectors, such as community pharmacies and private hospitals, in both urban and rural areas of Malaysia [14]. Hence, in the MPMS database, the same brand of a medicine referred to by the names given to them by their manufacturers (pharmaceutical companies) (e.g., Norvasc® is the brand name for the drug whose generic name is Amlodipine) may have a variety of prices from their multiple sampling sites. Because of that, the median price of the medicine brand was used for analysis in this study. Unlike the RRP medicine database which published to the public, the MPMS was only used by the PSD for monitoring purposes.

Only medicines listed in all three databases (the MPMS database; the database of medicine prices declared to PSD; and the International Reference Prices (IRP) database, which contains information on median buyer prices) [15] were included in this study. According to the brand names of the medicines, the following data were extracted from the databases: medicine prices, manufacturers’ category (local or imported), type of medicine (innovator brand [IB] [patented versions of the chemical entity first launched in the international market] or generic), classification (acute or chronic condition), location of MPMS sampling (e.g., Peninsular, East Malaysia, urban or rural area), and setting (community pharmacies or private hospitals). For all medicines, the unit drug price was calculated by dividing the drug-pack selling price by the number of tablets, capsules, vials, or doses it contained.

We obtained the Spearman correlation coefficient using the statistical package for the social sciences (SPSS) software (version 22.0; SPSS Inc., Chicago, IL, USA) to analyse the relationship between the prices declared to the PSD and retail medicine prices. The correlation test was performed for each year due to the limited number of medicines prices available throughout the 5-year period. *P* <  0.05 was considered statically significant. The difference between the retail prices, and the wholesale and RRP medicine prices was determined by the mean percentage difference. While the median price ratio (MPR) was calculated by comparing the median unit retail price to the median unit price in the IRP [14]. The IRP was converted to local currency using the average Bank Negara exchange rate for each year of the study. The following average USD exchange rates were used for 2011 to 2015: RM 3.00 (2011), RM 3.17 (2012), RM 3.19 (2012), RM 3.18 (2014), and RM 3.96 (2015). An MPR of 1 indicates the consumer price is equivalent to the IRP. In this study, an MPR ≤2.5 is considered as an acceptable retail medicine price in the private healthcare settings [16]. Retail medicine price trends were evaluated for medicines with pricing data reported continuously from 2011 to 2015. Using the Kruskal-Wallis test, the median retail price difference between the years was evaluated.

Factors that might influence the consumer retail prices with MPR > 2.5 were investigated using a binary logistic regression method with a backward likelihood-ratio analysis. Variables of interest such as settings, location, manufacturers’ category, type of dosage (tablet or others), originality, and price declared to the PSD were investigated, and those with *p* <  0.05 were considered to have significantly affected the consumer retail prices with MPR > 2.5. Prior to this evaluation, a univariate analysis was performed to determine the variables to be included in the final model analysis. Only those with *p* <  0.25 were included.

## Results

The yearly voluntary reporting of medicine prices by pharmaceutical companies was inconsistent. Based on the availability of medicine price data in the three databases, only 81 drug groups (e.g. Glibenclamide) and 237 brands (e.g. Daonil®) were included in the current study. Of these, the prices of only 15 medicine brands were consistently declared to the PSD from 2011 to 2015. Details of the medicines investigated in this study, presented according to their use (chronic and acute conditions), are mentioned in Additional file 1.

The wholesale price and declared RRP from 2011 to 2015 are significantly associated with the retail price (*p* <  0.05) (Table 1). For every year, all correlations were found to be strongly related, with correlation coefficients > 0.7. The percentage of medicine price markup from the declared PSD wholesale and wholesale price in MPMS to retail prices for 2011 to 2015 is presented in Table 2. The results showed that generic medicines always had a higher percentage of median price markups (53.1–110.8%) compared with the IB medicines (4.1–26.9%) (Table 2). Interestingly, markups of the wholesale medicine prices, deduced using MPMS retail prices, were always higher than the markups calculated from the wholesale price declared to the PSD.

**Table 1:** The relationship between prices declared to Pharmaceutical Service Division and retail medicine price

Year	Declared Wholesale price and retail medicine price		Declared recommended retail price (RRP) and retail medicine price	
	*Correlation coefficient, ρ*	*p-value*	*Correlation coefficient, ρ*	*p-value*
2011	0.92	*p* < 0.01	0.96	*p* < 0.01
2012	0.89	*p* < 0.01	0.89	*p* < 0.01
2013	0.92	*p* < 0.01	0.91	*p* < 0.01
2014	0.89	*p* < 0.01	0.89	*p* < 0.01
2015	0.75	*p* < 0.01	0.86	*p* < 0.01

**Table 2:** Percentage of medicine price markup from declared wholesale and retail wholesale to median retail price

Type of medicine	Year	Number of medicines brands reviewed	Number of pricing data from MPMS	Percentage price markup (%)					
				From declared wholesale^a^ to median retail price^b^			From median wholesale^b^ price to median retail price^b^		
				Median	25th percentile	75th percentile	Median	25th percentile	75th percentile
Innovator	2011	30	187	26.9	16.9	33.4	33.3	26.3	37.5
	2012	12	103	14.8	3.0	32.7	33.32	39.5	28.6
	2013	17	182	9.3	−6.3	29.6	38.9	33.3	45.8
	2014	10	176	18.3	−5.3	33.3	32.9	30.9	33.3
	2015	19	235	4.1	−2.9	32.8	33.4	29.4	47.1
Generic	2011	44	176	53.1	18.9	179.0	100.9	56.3	181.8
	2012	22	105	109.2	39.5	284.1	138.0	70.4	308.2
	2013	48	194	110.8	37.0	248.1	102.6	66.7	177.8
	2014	68	383	95.1	23.2	200.8	117.9	65.5	255.9
	2015	149	786	82.7	20.0	229.7	102.9	63.7	203.0

Note: *MPMS* Medicine Price Monitoring Survey

^a^Declared wholesale = the wholesale price declared by the pharmaceutical industries to Pharmaceutical Service Division (PSD), Ministry of Health Malaysia

^b^Median wholesale and retail price = medicine price sampled by the MPMS from various community pharmacy and private hospitals in Malaysia

A summary of the calculated MPR from 2011 to 2015 is shown in Table 3. The results show that most of the medicines included in the study had an MPR > 2.5. IB medicines were found to have a higher median MPR than their generic counterparts. The median MPR was found to be the highest in 2015 for both IBs (MPR 28.3) and generics (MPR 10.6). The 25th and 75th percentiles for IBs and generics were 8.0 and 40.4, and 4.2 and 18.1, respectively. Nevertheless, analysis of the retail medicine price for 15 medicine brands showed no significant increase in their retail medicine price for both IBs and generics throughout the 5-year period (*P* > 0.05) (Table 4). The model analysis (Table 5) revealed that the factors that might influence the medicines retail prices with MPR > 2.5 were: chronic disease use, locally manufactured, and sold in East Malaysia (*p* <  0.05). Moreover, generic medicines, medicines in rural areas, and medicines in other forms were less likely to have an MPR > 2.5. Interestingly an increase in the price of a single dose unit medicine RRP declared to the PSD by RM 0.01 (USD 0.003) increased the chances of a retail medicine having an MPR > 2.5 by 52% (OR, 1.52; 95% CI, 1.17 to 1.98; *p* < .05). Moreover, an RM 0.01 (USD 0.003) reduction in the retail wholesale price increased the chances of medicines having an MPR > 2.5 by 72% (OR, 0.72; 95% CI, 0.55 to 0.93; *p* = 0.013).

**Table 3:** Summary of median price range (MPR) of medicines in private healthcare sector in Malaysia from 2011 to 2015 (*n* = 2527)

Year	MPR > 2.5 n (%)	MPR ≤ 2.5 n (%)	Innovator Brand			Generic medicine		
			Median MPR	25th percentile	75th percentile	Median MPR	25th percentile	75th percentile
2011	337 (92.8)	26 (7.2)	20.2	6.4	37.7	8.7	4.5	16.2
2012	194 (93.3)	14 (6.7)	20.3	5.8	62.7	6.0	3.2	10.9
2013	368 (97.9)	8 (2.1)	25.2	4.4	39.6	7.4	4.6	11.6
2014	520 (93.0)	39 (7.0)	10.3	5.6	29.9	8.5	3.6	17.6
2015	906 (88.7)	115 (11.3)	28.3	8.0	40.4	10.6	4.2	18.1

**Note:** *MPR* Median Price Range

**Table 4:** The median retail price for 15 medicine brands from year 2011 to 2015

Year	MPMS retail price^a^ for sample of medicine from 2011 to 2015 (RM)														
	2011			2012			2013			2014			2015		
Generic name	Median	25th Per	75th Per	Median	25th Per	75th Per	Median	25th Per	75th Per	Median	25th Per	75th Per	Median	25th Per	75th Per
Innovator															
Hyoscine butylbromide 10 mg tablet	0.35	0.32	0.40	0.33	0.30	0.40	0.32	0.30	0.40	0.47	0.30	0.45	0.40	0.30	050
Glibenclamide 5 mg tablet	0.84	0.73	0.90	0.65	0.60	0.84	0.75	0.65	0.84	0.76	0.65	0.83	0.78	0.70	0.84
Gliclazide 80 mg tablet	1.33	1.10	1.51	1.24	1.00	1.50	1.17	1.08	1.50	1.20	1.01	1.50	1.42	1/17	1.80
Metformin 500 mg tablet	0.53	0.40	0.72	0.48	0.45	0.55	0.49	0.45	0.53	0.43	0.39	0.50	0.47	0.40	0.50
Frusemide 40 mg tablet	1.21	1.18	1.26	1.20	1.18	1.20	1.23	1.20	1.38	1.20	1.19	1.26	1.34	1.20	1.40
Generic															
Allopurinol 300 mg tablet	0.40	0.35	0.45	0.43	0.35	0.50	0.40	0.35	0.50	0.40	0.30	0.50	0.40	0.30	0.43
Isosorbide dinitrate 10 mg tablet	0.10	0.10	0.15	0.15	0.10	0.20	0.20	0.10	0.20	0.20	0.16	0.26	0.20	0.15	0.30
Amoxicillin 250 mg tablet	0.20	0.20	0.25	0.20	0.20	0.25	0.20	0.20	0.20	0.20	0.20	0.25	0.22	0.20	0.30
Doxycillin 100	0.42	0.35	0.50	0.30	0.30	0.50	0.30	0.30	1.00	0.38	0.23	0.73	0.48	0.38	0.75
Frusemide 40 mg tablet	0.30	0.30	0.35	0.20	0.10	0.30	0.30	0.30	0.30	0.30	0.15	0.50	0.40	0.12	0.50
Gliclazide 80 mg tablet	0.55	0.50	0.64	0.60	0.45	0.65	0.59	0.55	0.65	0.58	0.50	0.60	0.58	0.43	0.66
Prednisolone 5 mg tablet	0.18	0.12	0.25	0.20	0.20	0.20	0.20	0.20	0.25	0.20	0.20	0.20	0.20	0.16	0.20
Promethazine 1 mg/ml syrup	0.08	0.06	0.09	0.09	0.07	0.10	0.07	0.07	0.06	0.07	0.06	0.07	0.08	0.07	0.09
Loratadine 10 mg tablet	0.50	0.50	0.50	0.50	0.50	0.50	0.50	0.50	0.50	0.65	0.55	0.70	0.65	0.60	0.75
Simvastatin 20 mg tablet	1.00	1.00	1.00	0.83	0.63	1.00	1.00	0.67	1.00	1.00	1.00	1.00	0.50	0.50	0.50

^a^Retail prices were medicine price sampled through the Medicine Price Monitoring Survey (MPMS) from various sites such as community pharmacy and private hospitals in Malaysia; *RM* Ringgit Malaysia

**Table 5:** Factors that may influence medicines retail price of MPR > 2.5 in private healthcare sector in Malaysia

	MPR		Univariate analysis (*n* = 2527)			Multivariate analysis (*n* = 2527)		
	MPR ≤ 2.5 (*n* = 202), n (%)^a^	MPR > 2.5 (*n* = 2325), n (%)^a^	Crude OR (95% CI)	Wald’s χ2 (df)	P-value	Adj. OR (95% CI)	Wald’s χ2 (df)	P-value
Condition								
Acute	99 (11.3)	774 (88.7)	1.00			1.00		
Chronic	103 (6.2)	1551 (93.8)	1.93 (1.44, 2.57)	19.76 (1)	< 0.001	2.05 (1.46, 2.70)	16.84 (1)	< 0.001
Type of medicine								
Innovator brands	32 (3.6)	851 (96.4)	1.00			1.00		
Generic	170 (10.3)	1474 (89.7)	0.33 (0.22, 0.48)	32.22 (1)	< 0.001	0.24 (0.15, 0.37)	39.81 (1)	< 0.001
Manufacturer								
Import	149 (8.8)	1542 (91.3)	1.00			1.00		
Local	53 (6.3)	783 (93.7)	1.43 (1.03, 1.98)	4.61 (1)	0.032	3.86 (2.61,5.71)	45.88 (1)	< 0.001
Region								
Peninsular	161 (8.7)	1686 (91.3)	1.00			1.00		
East Malaysia	41 (6.0)	639 (94.0)	1.49 (1.04, 2.12)	4.83 (1)	0.028	1.84 (1.26, 2.68)	10.32 (1)	< 0.001
Type of Facility								
Community Pharmacy	179 (8.3)	1984 (91.3)	1.00					
Private Hospital	23(6.3)	341 (93.7)	1.34 (0.85, 2.09)	1.61 (1)	0.200	–	–	–
Location								
Urban	152(7.1)	1975 (92.9)	1.00			1.00		
Rural	50(12.5)	350 (87.5)	0.54 (0.38, 0.76)	12.77 (1)	< 0.001	0.48 (0.34, 0.69)	15.86 (1)	< 0.001
Form								
Tablet	161(6.9)	2181 (93.1)	1.00			1.00		
Other form	41(22.2)	144 (77.8)	0.26 (0.18, 0.38)	47.95 (1)	< 0.001	0.20 (0.13, 0.32)	51.53 (1)	< 0.001
PSD Wholesale	5.94 ^d^	15.70 ^e^	1.58 (1.27, 1.96)	17.29 (1)	< 0.001	–	–	NS
PSD RRP	8.60 ^d^	20.84 ^e^	1.47 (1.25, 1.74)	20.96 (1)	< 0.001	1.52 (1.17, 1.98)	10.03 (1)	< 0.001
MPMS Wholesale	5.21 ^d^	11.96 ^e^	1.36 (1.09, 1.69)	7.17 (1)	0.001	0.72 (0.55, 0.93)	6.10 (1)	0.013

Abbreviations: *MPR* median price range, *OR* odds ratio, *CI* Confidence interval, *NS* Non-significant, *PSD* Pharmaceutical Service Division, *RRP* Recommended Retail Price, *MPMS* Medicine PriceMonitoring Survey

**Notes:** ^a^ The percentage is reported by row, adding up to 100% based on available information; ^d^ Median price over IRP; ^e^ IQR of price over IRP

## Discussion

Voluntary medicine price declaration by pharmaceutical companies had a strong and significant association with private healthcare sector retail prices. Because the declared RRPs and median retail prices were significantly associated, the declared prices published in the PSD website can be considered reliable medicine pricing information for consumers. Accurate information on medicine prices is useful to consumers as it will provide them with options, thus, allowing for more efficient and effective procurement [12]. The latter may empower consumers and result in market competition, which may lead to reduced medicine retail price in the long run [12]. Meanwhile, the information about the significant association between the disclosure of wholesale medicine prices to the government and retail prices can be used for direct negotiations on agreed medicine prices with drug companies, as currently practiced in Canada and certain European countries [10, 11]. Nevertheless, only a few pharmaceutical companies declared their medicine prices to the PSD. Although various efforts have been made and numerous discussions have been held between government stakeholders and pharmaceutical industry representatives, price data for only 2527 medicines between 2011 and 2015 were available to be used in the study. Moreover, the data of only 15 medicine brands were consistently reported throughout the 5-year period. To increase the pharmaceutical industries practice of medicine price transparency, similar to South Africa, Vietnam, and some European countries, the government may want to include price disclosure in the pharmaceutical pricing regulations [17–19].

In this study, the median retail price of generic medicines had a higher percentage markup (up to 75% of their declared PSD wholesale price) than IB medicines. Nevertheless, when the retail prices were compared to the IRP, the median MPR for generic medicines was lower than that of IB. This shows that generic medicines in the private healthcare sector, although highly marked up, were more affordable than IB medicines. This is supported by the findings of Ahmad et al. [13], who reported that the affordability of 11 essential generic medicines reviewed in their study was better than that of IB medicines. In their study, treatment with generic medicines cost less than 2 days’ wages, while with IB, it cost 0.3 to 7.0 days’ wages [13]. The high median markup of retail prices may be attributable to the low baseline cost of generic medicines, offering an opportunity to gain higher profit margins, as reported in previous local and non-local studies [6, 20–22]. Furthermore, the increased likelihood of an MPR being > 2.5 with an RM 0.01 reduction in the wholesale price declared to the PSD may also be attributable to the same reasons. Although there is no medicine price control in the private healthcare sector in Malaysia, the median retail price for 15 medicine brands (5 IBs and 10 generics) was not significantly different throughout the 5-year period. Regardless, a majority of medicine prices in private facilities were found to have an MPR > 2.5.

The current study evaluated the constructs for medicine price in private healthcare settings with consideration on the price declaration practice by the pharmaceutical industries. The data included in the analysis involved large data set and many variables were investigated to evaluate the retail medicine prices which were analyzed as MPR. The MPR threshold of 2.5 was used in this study as an acceptable price markup following previous reported study in private healthcare sector [16]. Many factors contribute to making the MPR > 2.5. However, those worth our attention were location and type of manufacturer. The medicine price disparity between urban and rural areas may hinge on issues related to market segmentation. Segmentation can be welfare-enhancing, as suppliers are able to disaggregate the demand into segments of potential customers and charge according to willingness-to-pay. However, it may also result in price discrimination with some consumers being charged more than they are willing to pay [23]. This may affect the affordability of medicines to consumers and compromise their health. The disparity between medicine prices in urban and rural Malaysia could be overcome by introducing regulations on price markup along the pharmaceutical supply chain. The regulations must consider the additional overhead cost, distribution charges, and profits in urban and rural areas [24]. Regulating wholesale and retail price markup could prevent high medicine cost, as reported in Jordan, Kenya, Kuwait, and Lebanon [24].

Medicines from local manufacturers were more likely to have an MPR > 2.5 than those from foreign manufacturers. Although foreign manufacturers such as China and India are able to offer large volumes of medicines at low cost, supporting local manufacturers may be a better systemic approach for improving access to medicines [25]. Some of the advantages of having pharmaceuticals locally manufactured include faster delivery, ability to adapt medicines to local needs, and sufficient supply capacity in emergencies such as outbreaks. It also supports the country’s industrialization and creates new jobs. The higher medicine retail prices declared by local manufacturers may be attributed to the size of the companies. They are usually small and medium-sized companies that cater only to the domestic market, and higher profit margins are vital to their survival [5, 26]. To keep medicine prices competitive, the government may consider providing incentives to local manufacturers. For instance, in Vietnam, a national policy prioritising the purchase of domestically produced medicines has been imposed [27]. Other approaches include reducing corporate tax rates, providing soft loans, creating an investment-friendly environment, and increasing infrastructure development [28].

There are a few limitations to this study. First, the MPMS only included medicine prices sampled from community pharmacies and a limited number of private hospitals in Malaysia. It did not include the medicine prices from clinics of dispensing doctors. As such, the generalization of the present results to all distribution channels must be done cautiously. In addition, the medicine prices declared to the PSD did not disclose discount and bonus schemes. Since this type of procurement arrangement is usually confidential, it would be difficult to obtain true wholesale prices. In addition, the sample used for this study includes yearly medicine prices for the 5-year period for several pharmaceuticals, but the trend of retail pricing was only applicable to 15 medicine brands.

## Conclusion

The declaration of medicine prices was found to have a significant association with retail prices. Thus, the disclosed prices would serve as a useful reference for consumers when purchasing medicines in private healthcare settings. Several factors were found to influence the MPR of retail medicine prices, and could be addressed by several mechanisms. However, the government of Malaysia still must identify and implement strategies to increase medicine price transparency practice by pharmaceutical industry. This may include a regulatory policy that enforces price declaration and medicine price control mechanisms for the private healthcare sector. Results from research on medicine price transparency practices in other countries might provide useful information and guide policy regulation in Malaysia. In addition, future studies should explore the stakeholders’ views on the potential impact of policy regulations aimed at increasing medicine price transparency, price control, or both in the private healthcare sector in Malaysia.

## Additional file

Additional file 1: Appendix 1: List of medicines included in the study with indication for chronic and acute condition. (DOCX 26 kb)
