# Supplementary material for: Association between medicine Price declaration by pharmaceutical industries and retail prices in Malaysia’s private healthcare sector
Source: J Pharm Policy Pract. 2019 Jul 3;12:15. doi: 10.1186/s40545-019-0176-z (PMC6607540; doi:10.1186/s40545-019-0176-z)
Supplement: Supplementary file 1 — Appendix 1: List of medicines included in the study with indication for chronic and acute condition. (DOCX 26 kb) [file 40545_2019_176_MOESM1_ESM.docx]

**APPENDIX 1:** List of medicines included in the study with indication for chronic and acute condition

**List of medicines included in the study with indication for chronic condition**

| **No** | **Generic Name** | **No of brand** | | **No** | | **Generic Name** | | **No of brand** | |
| --- | --- | --- | --- | --- | --- | --- | --- | --- | --- |
| 1 | Amlodipine 10mg tablet/capsule | 8 | 24 | | Insulin neutral, soluble 100IU/ml penfill (3ml) | | 2 | |  |
| 2 | Amlodipine 5mg tablet/capsule | 9 | 25 | | Insulin neutral, soluble 100IU/ml vial (10ml) | | 1 | |  |
| 3 | Atenolol 100mg tablet/capsule | 5 | 26 | | Isosorbide dinitrate 10mg tablet/capsule | | 2 | |  |
| 4 | Atenolol 50mg tablet/capsule | 8 | 27 | | Isosorbide dinitrate 5mg tablet/capsule | | 1 | |  |
| 5 | Atorvastatin 20mg tablet/capsule | 3 | 28 | | Lisinopril 5mg tablet/capsule | | 1 | |  |
| 6 | Atorvastatin 40mg tablet/capsule | 1 | 29 | | Losartan 50mg tablet/capsule | | 2 | |  |
| 7 | Budesonide 200mcg/dose inhaler | 1 | 30 | | Lovastatin 20mg tablet/capsule | | 3 | |  |
| 8 | Captopril 25mg tablet/capsule | 2 | 31 | | Metformin 500mg tablet/capsule | | 5 | |  |
| 9 | Carbamazepine 200mg tablet/capsule | 2 | 32 | | Metoprolol 100mg tablet/capsule | | 2 | |  |
| 10 | Enalapril 10mg tablet/capsule | 3 | 33 | | Nifedipine 10mg tablet/capsule | | 1 | |  |
| 11 | Enalapril 20mg tablet/capsule | 3 | 34 | | Nifedipine retard 20mg tablet/capsule | | 1 | |  |
| 12 | Enalapril 5mg tablet/capsule | 2 | 35 | | Perindopril 4mg tablet/capsule | | 3 | |  |
| 13 | Fluoxetine 20mg tablet/capsule | 2 | 36 | | Phenytoin 100mg tablet/capsule | | 1 | |  |
| 14 | Frusemide 40mg tablet/capsule | 4 | 37 | | Prazosin 1mg tablet/capsule | | 1 | |  |
| 15 | Glibenclamide 5mg tablet/capsule | 3 | 38 | | Salbutamol 100mcg/dose inhaler | | 3 | |  |
| 16 | Gliclazide 80mg tablet/capsule | 5 | 39 | | Salbutamol 2mg tablet/capsule | | 1 | |  |
| 17 | Hydrochlorothiazide 25mg tablet/capsule | 1 | 40 | | Ranitidine 150mg tablet/capsule | | 7 | |  |
| 18 | Hydrochlorothiazide 50mg tablet/capsule | 2 | 41 | | Ranitidine 300mg tablet/capsule | | 1 | |  |
| 19 | Indapamide 2.5mg tablet/capsule | 3 | 42 | | Simvastatin 10mg tablet/capsule | | 8 | |  |
| 20 | Insulin biphasic 30/70 100IU/ml penfill (3ml) | 1 | 43 | | Simvastatin 20mg tablet/capsule | | 8 | |  |
| 21 | Insulin biphasic 30/70 100IU/ml vial (10ml) | 1 | 44 | | Simvastatin 40mg tablet/capsule | | 3 | |  |
| 22 | Insulin isophane (NPH) 100IU/ml penfill (3ml) | 2 | 45 | | Sodium valproate 200mg tablet/capsule | | 1 | |  |
| 23 | Insulin isophane (NPH) 100IU/ml vial (10ml) | 1 |  | |  | |  | |  |

**List of medicines included in the study with indication for acute condition**

| **No** | **Generic Name** | **No of brand** | **No** | **Generic Name** | | **No of brand** |  |
| --- | --- | --- | --- | --- | --- | --- | --- |
| 1 | Acetylsalicylic acid 300mg tablet/capsule | 3 | 19 | | Diazepam 5mg tablet/capsule | 2 | |
| 2 | Acetylsalicylic acid, glyprin (100+45) mg tablet/capsule | 1 | 20 | | Diclofenac 25mg tablet/capsule | 1 | |
| 3 | Aciclovir 200mg tablet/capsule | 2 | 21 | | Diclofenac 50mg tablet/capsule | 4 | |
| 4 | Albendazole 200mg tablet/capsule | 3 | 22 | | Doxycycline 100mg tablet/capsule | 4 | |
| 5 | Allopurinol 300mg tablet/capsule | 3 | 23 | | Gentamicin 0.3% eye drop | 1 | |
| 6 | Amitriptyline 25mg tablet/capsule | 1 | 24 | | Hyoscine butylbromide 10mg tablet/capsule | 4 | |
| 7 | Amoxicillin 250mg tablet/capsule | 8 | 25 | | Ibuprofen 200mg tablet/capsule | 4 | |
| 8 | Amoxicillin 25mg/ml suspension | 4 | 26 | | Ibuprofen 400mg tablet/capsule | 4 | |
| 9 | Amoxicillin 500mg tablet/capsule | 2 | 27 | | Lactulose 0.67g/ml syrup | 2 | |
| 10 | Amoxicillin 50mg/ml suspension | 1 | 28 | | Loratadine 10mg tablet/capsule | 7 | |
| 11 | Amoxicillin, clavulanic acid (500+125)mg tablet/capsule | 1 | 29 | | Mefenamic acid 250mg tablet/capsule | 2 | |
| 12 | Ceftriaxone 1g powder for injection | 2 | 30 | | Mefenamic acid 500mg tablet/capsule | 3 | |
| 13 | Cephalexin 250mg tablet/capsule | 1 | 31 | | Metronidazole 200mg tablet/capsule | 2 | |
| 14 | Cetirizine 10mg tablet/capsule | 5 | 32 | | Omeprazole 10mg tablet/capsule | 1 | |
| 15 | Chlorpheniramine 4mg tablet/capsule | 7 | 33 | | Omeprazole 20mg tablet/capsule | 8 | |
| 16 | Ciprofloxacin 500mg tablet/capsule | 1 | 34 | | Paracetamol 24mg/ml suspension | 1 | |
| 17 | Clotrimazole 1% cream | 2 | 35 | | Prednisolone 5mg tablet/capsule | 5 | |
| 18 | Co-trimoxazole (8+40)mg/ml suspension | 1 | 36 | | Promethazine 1mg/ml syrup | 4 | |
